# Supplementary material for: Optimal Techniques for EUS-Guided Fine-Needle Aspiration of Pancreatic Solid Masses at Facilities without On-Site Cytopathology: Results from Two Prospective Randomised Trials
Source: J Clin Med. 2021 Oct 12;10(20):4662. doi: 10.3390/jcm10204662 (PMC8540534; doi:10.3390/jcm10204662)

**Supplementary Figure S1.** Two types of 10 mL suction syringes were applied randomly and the operator was blind to whether suction was used or not during EUS-guided fine needle aspiration. **(A)** True suction syringe was prepared as the piston was pulled down in the closed state of valve. **(B)** False suction (nonsuction) syringe was prepared as the the piston was pulled down in the open state of valve and then the valve was closed later.

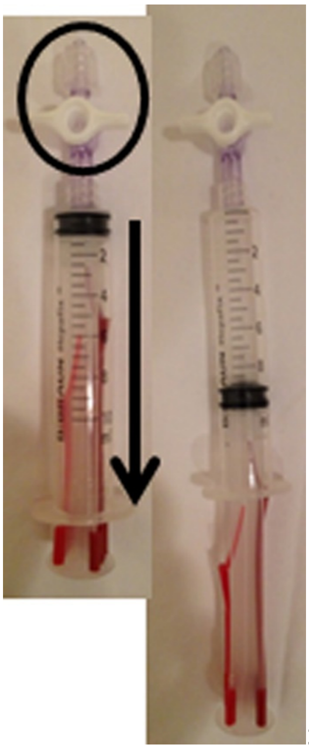

Supplementary Figure S1A

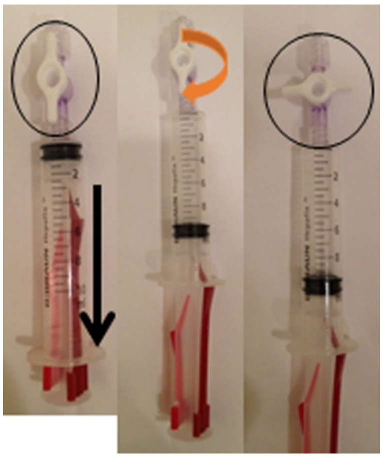

Supplementary Figure S1B

**Supplementary Figure S2.** A bench test showed capillary sampling with stylet slow-pull had -2 in.Hg and 10ml negative suction had -20.5 in.Hg (in.Hg: A unit of measure for pressure or vacuum, used to indicate how high a column of mercury can be pushed by pressure within a sealed tube).

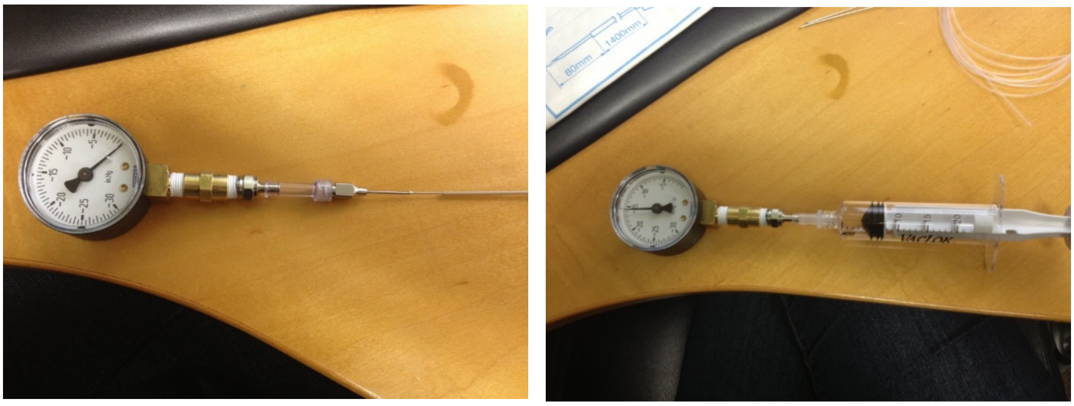

Supplement: Supplementary file 1 [file jcm-10-04662-s001.zip › jcm-1315905-supplementary.pdf]
